# Validation of Reference Genes for Real-Time PCR of Reproductive System in the Black Tiger Shrimp

**DOI:** 10.1371/journal.pone.0052677

**Published:** 2012-12-28

**Authors:** Rungnapa Leelatanawit, Amornpan Klanchui, Umaporn Uawisetwathana, Nitsara Karoonuthaisiri

**Affiliations:** 1 Microarray Laboratory, National Center for Genetic Engineering and Biotechnology, National Science and Technology Development Agency, Klong Luang, Pathumthani, Thailand; Kyushu Institute of Technology, Japan

## Abstract

Gene expression of reproductive system of the black tiger shrimp (*Peneaus monodon*) has been widely studied to address poor maturation problem in captivity. However, a systematic evaluation of reference genes in quantitative real-time PCR (qPCR) for *P. monodon* reproductive organs is lacking. In this study, the stability of four potential reference genes (*18s rRNA*, *GAPDH*, *β-actin*, and *EF1-α*) was examined in the reproductive tissues in various conditions using bioinformatic tools: NormFinder and geNorm. For NormFinder, *EF1-α* and *GAPDH* ranked first and second as the most stable genes in testis groups whereas *GAPDH* and *EF1-α* were for ovaries from wild-caught broodstock and domesticated groups. *EF1-α* and *β-actin* ranked first and second for the eyestalk ablated ovaries. For geNorm, *EF1-α* and *GAPDH* had the best stability in all testis and ovaries from domesticated groups whereas *EF1-α* and *β-actin* were the best for ovaries from wild-caught and eyestalk ablated groups. Moreover, the expression levels of two well-known reproductive genes, *Dmc1* and *Vitellogenin*, were used to validate these reference genes. When normalized to *EF1-α,* the expected expression patterns were obtained in all cases. Therefore, this work suggests that *EF1-α* is more versatile as reference genes in qPCR analysis for reproductive system in *P. monodon*.

## Introduction

Quantitative real-time polymerase chain reaction (qPCR) is a useful technique to measure gene expression levels due to its high sensitivity, accuracy, and reproducibility. To employ qPCR for gene expression analysis, housekeeping genes are used as internal control to normalize expression levels of other genes of interest. Therefore, it is important to select a reference gene whose expression level is constitutive and constant under different experimental conditions or biological samples for a particular study.

Several internal control genes have been validated for qPCR in different experimental conditions in many organisms such as human tissues [1], [2], [3], [4], [5], *Pimephales promelas* [6], *Oryzias latipes* [7], *Solea senegalensis* and *Hippoglossus hippoglossus* [8], *Danio rerio* [9], rice *Oryza sativa* L. ssp. *Indica* var. IR64 [10], soybean *Glycine max* [L.] Merr. [11], and *Leptospira* [12]. In the Pacific blue shrimp *Penaeus stylirostris*, *elongation factor 1 alpha* (*EF-1α*) and *glyceraldehydes-3 phosphate dehygrogenase* (*GAPDH*) have been validated as reference genes for expression analysis of immune genes [13]. In the black tiger shrimp (*P. monodon),* several housekeeping genes, such as *β-actin* [14], [15], [16], *EF-1α* [*17*], [18], [19], [*20*], *GAPDH* [19], *40S rRNA* [21], *18S rRNA* [16], [22], and *elongation factor 2* [23] have been used as an internal control for qPCR. However, to date, no study has validated their suitability as an internal control for gene expression analysis using qPCR in *P. monodon*.

Recently, the reproductive system of both male and female *P. monodon* has been extensively studied because poor reproductive maturation in captivity presents a serious threat to the shrimp farming industries. Although several studies employed qPCR to examine gene expression profiles during reproductive maturation [18], [20], [24], [25], [26], [27], [28], the gene expression studies for the reproductive system of this organism can be inaccurate without using appropriate internal control genes. In this study, we validated four commonly used reference genes (*18S rRNA*, *GAPDH*, *β-actin,* and *EF-1α*) to be used as an internal control in qPCR analysis of reproductive samples with various conditions. Gene expression levels of these four genes in three ovary sample groups (wild-caught broodstock, domesticated shrimp, and eyestalk ablated broodstock) and two testis sample groups (wild-caught broodstock and domesticated shrimp) were measured by qPCR and two computational analysis tools (geNorm and NormFinder) were used to compare expression stability of the four candidate reference genes. Moreover, relative expression levels of two reproductive genes, *Dmc1* for testicular development [25] and *Vitellogenin* for ovarian maturation [29], [30], were also measured using the four candidate reference genes for normalization.

## Results and Discussion

### Expression Levels of Housekeeping Genes in Reproductive Organ of *Penaeus monodon* by Quantitative Real-time PCR (qPCR)

Due to its accuracy, sensitivity, fast speed and reproducibility, quantitative real-time PCR (qPCR) has become a useful method for gene expression analysis. However, its accuracy relies upon a good reference gene whose expression levels should remain stable across tissues and different environmental conditions. Nevertheless, there is no an ultimate gene to be used as an internal control for all cell types or all experimental conditions. For gene expression analysis of the reproductive system in the black tiger shrimp *Peneaus monodon*, samples differ between individuals, tissues, growth stages and developmental stages; yet no previous study has examined the most appropriate genes to be used as an internal control gene.

**Figure 1:**
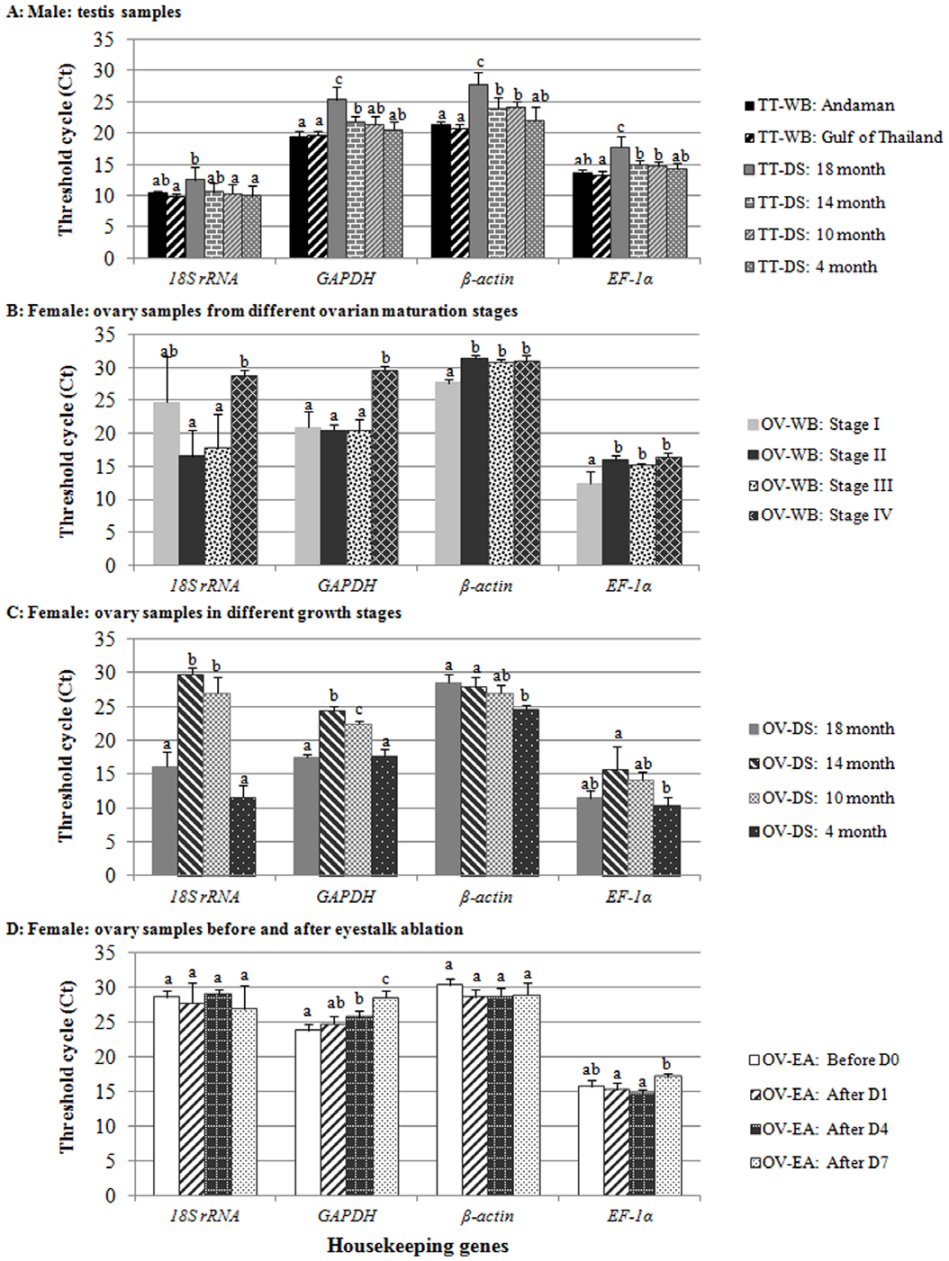
Threshold cycle values (Ct) of four housekeeping genes (*18s rRNA*, *GAPDH*, *β-actin*, and *EF1-α*) determined by qPCR from (A) testis samples of wild broodstock from different locations (Andaman sea and Gulf of Thailand) and domesticated shrimp with different growth stages (18-, 14-, 10-, and 4-month-old domesticated shrimp, DS), (B) ovary samples from wild broodstock with different ovarian maturation stages (Stages I–IV), (C) ovary samples of domesticated shrimp from different growth stages (18-, 14-, 10-, and 4-month-old domesticated shrimp, DS), and (D) ovary samples from 14-month-old domesticated broodstock before and after eyestalk ablation for 1, 4 and 7 days. Different letters above the bars signify statistical differences.

In this study, four commonly used housekeeping genes (*18S rRNA*, *GAPDH*, *β-actin*, and *EF-1α*) in qPCR gene expression analysis were validated for their suitability as a reference gene for reproductive organs of *P. monodon*. Male samples tested in this study were testes (TT) of wild brooders (WB) from Andaman Sea and Gulf of Thailand and of domesticated shrimp (DS) at 4-, 10-, 14-, and 18-month-old). Female samples were ovaries (OV) from wild brooders (WB) with various degree of reproductive maturation (Stages I–IV), from domesticated shrimp (DS) at 4-, 10-, 14-, and 18-month-old, and from domesticated broodstock before and after eyestalk ablation (EA; an eyestalk ablation is a common practice to induce ovarian maturation) at day 1, 4, and 7 (Table 1). To determine expression profiles of these housekeeping genes in the shrimp reproductive system, threshold cycle (Ct) values of all sample groups were measured (Fig. 1).

**Table 1:** Testis and ovary samples from *P. monodon* used in this study.

No.	Samples	GSI*
***Ovary samples***		
**I**	**Wild broodstock (OV-WB)**	
	Stage I	*n* = 5; GSI = 0.69±0.27%
	Stage II	*n* = 5; GSI = 2.45±0.32%
	Stage III	*n* = 5; GSI = 4.80±1.12%
	Stage IV	*n* = 5; GSI = 11.43±1.29%
**II**	**Domesticated shrimp (OV-DS)**	
	18-month-old domesticated shrimp (DS)	*n* = 7; GSI = 0.88±0.29%
	14-month-old DS	*n* = 5; GSI = 1.04±0.29%
	10-month-old DS	*n* = 5; GSI = 0.66±0.30%
	4-month-old DS	*n* = 5; GSI = NA
**III**	**Eyestalk ablated 14-month-old domesticated broodstock** **(OV-EA)**	
	Before eyestalk ablation: Day 0 (D0)	*n* = 5; GSI* = 1.14±0.17%
	After eyestalk ablation: Day 1 (D1)	*n* = 5; GSI* = 1.22±0.14%,
	Day 4 (D4)	*n* = 5; GSI* = 1.31±0.33%
	Day 7 (D7)	*n* = 5; GSI* = 5.18±2.31%
***Testis samples***		
**I**	**Wild broodstock (TT-WB)**	
	Andaman Sea (West)	*n* = 5; GSI = 1.14±0.26%
	Gulf of Thailand (East)	*n* = 5; GSI = 0.74±0.12%
**II**	**Domesticated shrimp (TT-DS)**	
	18-month-old domesticated shrimp (DS)	*n* = 7; GSI = 0.53±0.21%
	14-month-old DS	*n* = 5; GSI = 0.51±0.05%
	10-month-old DS	*n* = 5; GSI = 0.69±0.25%
	4-month-old DS	*n* = 5; GSI = NA

* GSI is gonadosomatic index calculate as a percentage of testis weight by total body weight.

In all testis sample groups, the expression patterns of four housekeeping genes were similar with the significantly higher Ct values found in 18-month-old domesticated broodstock (TT-DS: 18 month) than the other groups. *GAPDH* and *β-actin* genes showed similar expression levels ranging from 20–30 cycles, whereas *18S rRNA* and *EF-1α* were expressed lower from 10–20 cycles (Fig. 1A).

In the female group, the ovary samples were categorized into three groups: different ovarian maturation stages, different growth stages, and before and after eyestalk ablation (Figs. 1B–1D). For different ovarian maturation stages, two distinct expression patterns were observed. The *β-actin* and *EF-1α* expression patterns showed the similar trend with the lowest levels in Stage I and became higher but at a constant level during Stages II–IV. In contrast, the expression profiles of *18S rRNA* and *GAPDH* showed different patterns with higher variation in expression levels throughout different stages (Fig. 1B). For different growth stages, similar expression profiles of *18S rRNA*, *GAPDH*, and *EF-1α* were observed but *EF-1α* has the lowest variation of expression levels among these three genes. Although *β-actin* had a distinct pattern from the rest, its expression levels throughout growth stages were more constant (Fig. 1C). In the case of ovaries from non-ablated and ablated broodstock, the expression profiles of all housekeeping genes were similar, except for that of *GAPDH* whose levels were significantly different after the eyestalk ablation for 7 days (Fig. 1D).

In addition, when the expression profiles (Ct) of the four housekeeping genes were compared in all sample groups, *18S rRNA* and *GAPDH* showed high variation of the Ct values ranging from 10–30 cycles, while *β-actin* (Ct = 25–30 cycles) and *EF-1α* (Ct = 10–15 cycles) were expressed with less variation. Although *GAPDH*, an important gene encoding for a glycolytic pathway enzyme in carbohydrate metabolism, was frequently used as an internal control for qPCR analysis, it seems to be a good internal control only for lowly expressed genes [31]. Some studies showed *GAPDH* was unsuitable as an internal control due to its significant variation of expression levels between different individuals during pregnancy [32], with developmental stages [33], [34] and during the cell cycle of human cells [35], which agrees with our result when mRNA from different individuals and developmental stages were examined. For the case of *18S rRNA*, this ribosomal subunit gene was previously used as internal control in the gene expression studies of rice with environmental stresses [36] and the fathead minnow fish with environmental estrogens exposure [37]. However, there are two main drawbacks that *18S rRNA* cannot be used for normalization: (1) *rRNA* can be lost during mRNA purification, and (2) it is expressed at much greater levels than target mRNAs [38]. Perhaps, the biological functions of proteins encoded by *GAPDH* and *18S rRNA* suggest that their transcript levels are significantly regulated by various experimental settings and variable in different tissues and thus unstable [39], [40].

Unlike *GAPDH* and *18S rRNA*, the expression levels of *EF-1α* and *β-actin* were found to be more stable with lower variation in threshold cycles (Ct) in this study. Considering the Ct values, *EF-1α* is more suitable for normalization than *β-actin* because of its lower threshold cycle than that of *β-actin*. As a matter of fact, *β-actin,* encoding a cytoskeletal protein, was previously reported to have wide variation in its transcript levels in response to experimental manipulation in human breast epithelial cells [41], and blastomeres [42], as well as in various porcine tissues [43] and canine myocardium [44]. Its expression levels also varied in sample sets from embryonic, larval, and post-larval stages and gonad of the Kuruma shrimp [45]. In addition, the presence of *β-actin* pseudogenes can interfere with the interpretation of expression results as the same primer will detect both *β-actin* mRNA and DNA from this pseudogene [46]. For *EF-1α*, this transcriptional factor gene was employed as an internal control gene in gene expression studies of different tissues from the Atlantic salmon [47], [48], samples from different developmental conditions in the desert locust [49], and samples during larval development in the flatfish [50]. *EF-1α* was also the most suitable internal control for measuring the highly expressed genes [13].

### Stability in Expression Levels of the Housekeeping Genes

To systematically examine the stability in expression levels of the four housekeeping genes, two computational methods were employed: NormFinder and geNorm. The Ct values of each gene in both testis and ovary samples were converted into copy numbers using their standard curves. The algorithms of both methods aim to identify genes whose expression levels are most stable by assigning the highest stability value for the maximum number of time points.

**Figure 2:**
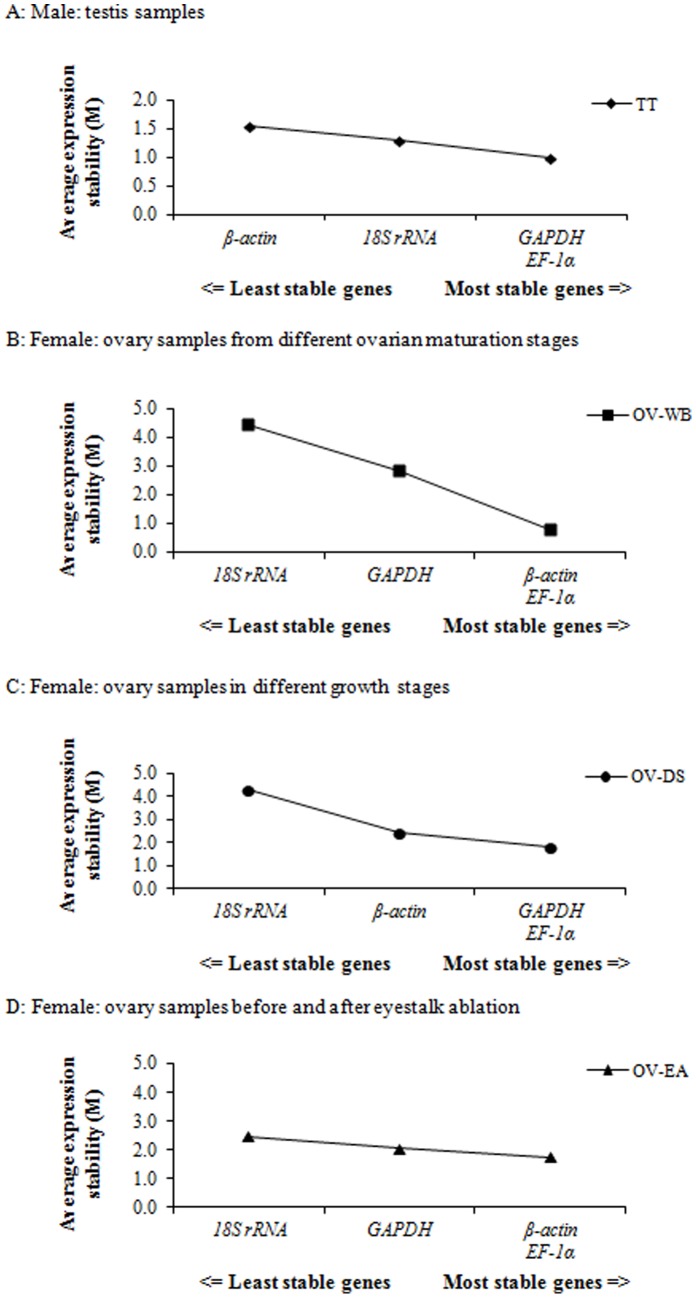
Average expression stability values (M), which is the mean pair-wise variation between an individual gene and all other tested genes, determined by geNorm software. (A) Average M value of 46 testis samples in *P. monodon* (TT), (B) Average M value of ovary samples from wild broodstock with different ovarian maturation stages (OV-WB), (C) Average M value of ovary samples from domesticated shrimp with different growth stages (OV-DS) and (D) Average M value of ovary samples from domesticated broodstock before and after eyestalk-ablation (OV-EA) in *P. monodon*.

The first method, geNorm, was used to calculate an average expression stability values (M values) by averaging pair-wise variation of a particular gene across all examined reference genes. It allows the most appropriate reference gene to be chosen by using the geometric mean of the expression of the candidate cDNA [51]. However, the program provides the final result as the two most stable genes for a multivariate data set. As a result, the two most stable genes with the lowest M value were given. For testis samples (TT) and female domesticated shrimp with different growth stages (OV-DS), *GAPDH* and *EF-1α* genes had the lowest M values suggesting most stable expression levels (Fig. 2A and 2C), whereas *β-actin* and *EF-1α* genes were the most stable pair for OV-WB and OV-EA groups (Fig. 2B and 2D). Another method, NormFinder, was separately used to confirm the results from geNorm. Not only does it measure the variation of expression levels, but it also ranks potential reference genes by how much they differ between study groups; in another word, it measures the extent by which they are affected by the experimental conditions [52]. It estimates the expression variation among candidate genes using a model-based approach to calculate a stability value for each gene and identify the single best reference gene with the highest stability in expression level indicated by the lowest value. Stability values and ranking order of the candidate reference genes in a given sample group were shown in Table 2. In the testis samples (TT) and ovary samples during the eyestalk ablation (OV-EA), *EF-1α* has the best stability value, whereas *GAPDH* has the best value for female wild broodstock from different ovarian maturation stages (OV-WB) and domesticated shrimp from different growth stages (OV-DS). However, the *GAPDH* stability was lowest in samples from the eyestalk ablation experiment.

**Table 2:** Stability values and ranking order (in parentheses) of the candidate reference genes measured by the NormFinder software.

	Stability Values			
Genes	Male		Female	
	(TT)	OV-WB	OV-DS	OV-EA
***18S rRNA***	1.05 (3)	4.08 (4)	3.97 (4)	1.31 (3)
***GAPDH***	0.34 (2)	1.16 (1)	0.79 (1)	1.37 (4)
***β-actin***	1.09 (4)	2.19 (3)	1.91 (3)	1.07 (2)
***EF-1α***	0.09 (1)	1.63 (2)	1.23 (2)	0.55 (1)

The genes with the highest stability values were hightlighted in each case. TT = testis samples, OV-WB = ovary samples from different ovarian maturation stages, OV-DS = ovary samples in different growth stages, and OV-EA = ovary samples before and after eyestalk ablation.

When compare between two methods, we found that *EF-1α* appeared to have most stable expression levels for testis samples (TT) and ovary samples during the eyestalk ablation (OV-EA). The only sample group from wild broodstock ovaries (OV-WB) and female domesticated shrimp with different growth stages (OV-DS) gave varied result. Although the most stable genes from NormFinder method found in OV-WB and OV-DS groups belonged to *GAPDH*, the second rank of stable genes belonged to *EF-1α* which correlated to the most stable genes from geNorm method. From geNorm method, *EF-1α* was only one gene found to be the most stable genes in all four sample groups (TT, OV-WB, OV-DS, and OV-ES). Moreover, *EF-1α* was in the first and second ranks of stable gene from NormFinder method, whereas *GAPDH* and *β-actin* ranked the forth (OV-EA) and the third (OV-WB and OV-DS) of stable gene, respectively. Therefore, the appropriate internal control for reproductive system of *P. monodon* seemed to be *EF-1α* due to its most stable expression levels across samples.

One caution to be considered is that both software algorithms rely upon an assumption that the expression of these reference genes should remain constant across the sample groups. However, this might not be the case in all conditions. Therefore, it is noteworthy to also consider other algorithms based on normalization software tools in this type of evaluation [53], [54].

### Validation of Housekeeping Genes with Reproductive-relevant Genes in Reproductive System in *P. monodon*

To validate whether these housekeeping genes are suitable as internal controls for qPCR analysis of reproductive gene expression in *P. monodon*, they were used as a reference gene for expression analysis of two known reproductive-relevant genes (*Dmc1* for testis and *Vg* for ovary) whose expression patterns were previously reported. The relative expression value and the absolute copy number were measured from standard curves of *Dmc1* and *Vg* using these reference genes.

**Figure 3:**
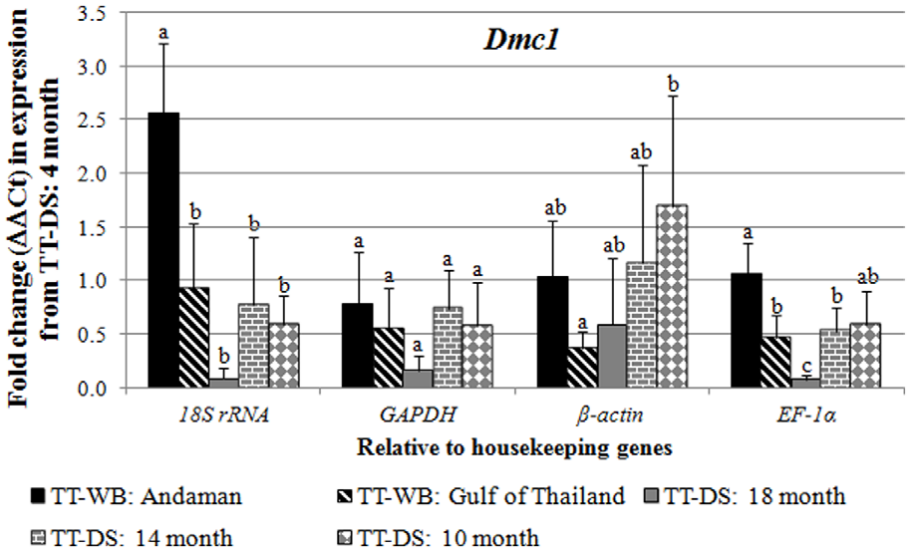
Relative expression levels in term of fold change (ΔΔCt) of a known testis-relevant marker, *Dmc1*, to those of housekeeping genes in 5 testis sample groups; wild broodstock from Andaman sea (black), wild broodstock from Gulf of Thailand (diagonal lines), 18-month-old domesticated shrimp (DS) (gray), 14-month-old DS (horizontal lines), and 10-month-old DS (diamond), were compared with four housekeeping genes (*18s rRNA*, *GAPDH*, *β-actin*, and *EF1-α*) in 4-month-old domesticated shrimp. Different letters above the bars of each graph signify statistical differences in gene expression levels within the sample group.

*Dmc1*, a RAC A-like recombinase, is known to be a specific factor for meiotic recombination and has been identified as a molecular marker for initial stages of meiosis because it was specifically expressed during the early meiotic prophase [55]. Moreover, *Dmc1* is reportedly to be essential for meiosis as found in several species such as humans [56], mice [56], mouse [57], Japanese eel (*Anguilla japonica*) [58], whiteleg shrimp (*Litopenaeus vannamei*) [59], *Caenorhabditis elegans*, [60], rice (*Oryza sativa* L. ssp. *japonica*) [61], *Arabidopsis thaliana* [62], and yeast [63]. It was also used as a gene marker for particular purposes; for example, for spermatocyte-specific gene for study the role of sumoylation in *vivo* in mice and mouse [64], for comparing gene expression profile of *TOPAZ1*, which is potential marker for germ cell development [65], and for study of social status and gonadotropic signals on testis development in Nile tilapia (*Oreochromis niloticus*) [66]. Moreover, *Dmc1* was also discovered in testis cDNA library of Crustacea, mitten crab (*Eriocheir sinensis*) [67], and *P. monodon* [26]. Furthermore, the expression levels of *Dmc1* were correlated to testis maturation degrees in the *P. monodon* and it was also proposed to be an indicator for early stages of germ cell development in *L. vannamei* [25], [59]. Therefore, the *Dmc1* expression level was normalized to each candidate genes to examine their suitability as an internal reference (Fig. 3). Using *18S rRNA*, *GAPDH*, and *EF-1α* as reference genes, the *Dmc1* exhibited similar profile as previously reported with the highest expression levels found in testis of wild broodstock from Andaman sea (TT-WB:Andaman) and the lowest found in testis of 18-month-old TT-DS while the rest of samples were expressed equally [25]. Although *EF-1α* was also used as a reference gene in the previous report and the both expression profiles of *Dmc1* were similar, the samples used in both experiments were completely different demonstrating robustness of *EF-1α* as an internal reference. Moreover, when consider the fold change in expression of the samples relatively to that of 4-month-old TT-DS, statistical analysis indicated that the expression levels normalized to *EF-1α* gave significant differences between testis groups with lower variation than those normalized to *18S rRNA* and *GAPDH*. On the other hand, the *Dmc1* expression pattern normalized to *β-actin* exhibited a different pattern from the others (Fig. 3). Likewise, the expression pattern and significant level of the genes in copy number suggested the same results as in the fold change (Fig. S1).

**Figure 4:**
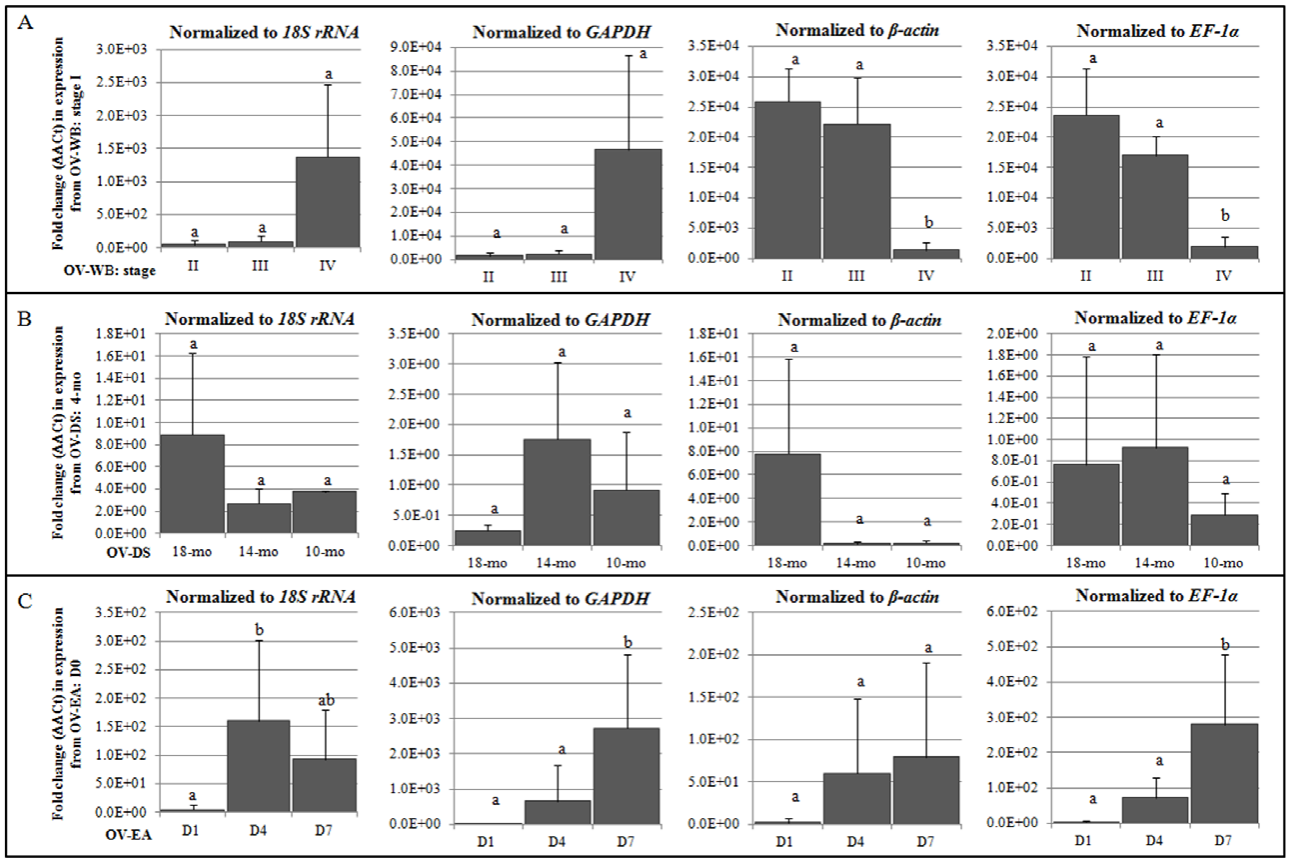
Relative expression levels in term of fold change (ΔΔCt) of a known ovary-relevant marker, *Vitellogenin* (*Vg*), to those of the housekeeping genes in three ovary sample groups: **(A) Wild broodstock (WB) from four different ovarian maturation stages compared to those of the housekeeping genes in WB stage 1, (B) Domesticated shrimp at 18-month-, 14-month-, and 10-month-old compared to those of the housekeeping genes in domesticated shrimp at 4-month-old, (C) Domesticated broodstock after the ablation for 1 (D1), 4 (D4), and 7 (D7) days compared to those of the housekeeping genes in before the ablation (D0).** Different letters above the bars of each graph signify statistical differences in gene expression levels within the sample group.

*Vitellogenin* (*Vg*) is a well-known indicator for ovarian maturation indicated by higher values in gonadosomatic index (GSI, ratio between gonad weight to body weight indicating ovarian maturation degrees) [68]. Based on the GSI value, ovarian maturation in penaeid shrimp can be categorized into four stages (I–IV): pre-vitellogenic, vitellogenesis, cortical rod, and late cortical rod [69]. Previous reports showed that the *Vg* expression level was low at previtellogenic stage (Stage I), increased to the highest level at vitellogenic stage (Stage II) and slightly decreased at early cortical rod (Stage III) and late cortical rod (Stage IV) in Kuruma prawn [30], [70] and Banana shrimp [29]. In our study, the fold change in the *Vg* expression during different maturation stages (OV-WB) relative to Stage I when normalized to *EF-1α* and *β-actin* showed similar profiles to the previously reports with significantly higher expression levels during Stages II–IV [29], [30], [70]. In contrast, the opposite expression trend was observed when normalized to *GAPDH* and *18S rRNA*. In OV-DS group, no significant difference in expression level of *Vitellogenin* was observed which could be explained from their GSI values that belong to Stage I for all the samples. Therefore, it would be difficult to validate the housekeeping genes with this sample group where there is no previous report on the *Vg* expression pattern during growth or different age in domesticated shrimp. For the samples from eyestalk ablation experiment (OV-EA), GSI values of Day 1 and Day 4 samples belong to Stage I (1.22±0.14% and 1.31±0.33%, respectively), while GSI value of Day 7 samples belongs to Stage III (5.18±2.31%). The *Vg* expression pattern normalized to *EF-1α*, *GAPDH* and *β-actin* showed similar pattern which agreed with the previous report that suggested increasing expression levels of *Vg* after an eyestalk ablation. Only the *Vg* expression levels normalized to *EF-1α* or *GAPDH* exhibited significantly higher levels in the Day 7 samples which were accordant to previous reports [29], [30], [70]. Besides, the *Vg* expression pattern normalized to *18S rRNA* showed a different profile with no correlation to previous reports (Fig. 4). The expression pattern and significant level of the genes in copy number suggested the same results as in the fold change (Fig. S2).

When *EF-1α* was used, the obtained expression patterns of both testicular development marker (*Dmc1*) and ovarian maturation marker *(Vg*) agreed with the previously report to the levels of statistically significant differences in most of the cases [18], [20], [25], [29], [30].

In conclusion, an appropriate choice of an internal control gene in relative quantification for reproductive gene expression profile in the black tiger shrimp is clearly important and needed to be carefully evaluated for their robustness. We identified the most stable reference genes for qPCR gene expression analysis by comparing the stability of commonly used reference genes using two bioinformatic programs, geNorm and NormFinder. *EF-1α* was validated to be the most reliable internal control gene for qPCR gene expression analysis of reproductive system in the black tiger shrimp. The result from this study will help future gene expression studies to use an appropriate internal control gene to avoid bias and inaccurate result.

## Materials and Methods

### Ethics Statement

No specific permits were required for the described field studies. The field studies did not involve endangered or protected species.

### RNA Samples and Reverse Transcription

Testis and ovary sample groups from male and female *P.monodon* were examined in this study. Testes samples were collected from wild broodstock (TT-WB) from Andaman Sea and Gulf of Thailand: West and East and domesticated shrimp at different growth stages (TT-DS: 4-, 10-, 14-, and 18-month-old). Ovary samples were collected from wild broodstock with different ovarian maturation stages (OV-WB: Stages I–IV), domesticated shrimp with different growth stages (OV-DS: 4-, 10-, 14-, and 18-month-old) and domesticated broodstock before and after eyestalk ablation for 1, 4 and 7 days (OV-EA: D0, D1, D4, and D7) as shown in Table 1. All samples were quickly frozen in liquid nitrogen for RNA extraction. RNA samples were extracted from the tissues using TRI-REAGENT according to manufacturer’s instruction (Molecular Research Center, USA). Contaminated genomic DNA was removed by treatment with DNase I at 0.15 U/µg total RNA at 37°C for 30 min. One microgram of total RNA was reverse transcribed (RT) using RevertAid™ First Strand cDNA Synthesis Kits (Fermentas) for testis samples and ImProm-II™ Reverse Transcription System (Promega) for ovary samples according to manufacturer’s instructions. The quantity of cDNA was measured using NanoDrop (ND-8000).

### Quantitative Real-time PCR (qPCR)

The expression levels of four housekeeping genes (*18S rRNA*, *GAPDH*, *β-actin,* and *EF-1α*) and testis-relevant (*Dmc1)* and ovary-relevant (*Vitellogenin)* transcripts in different shrimp conditions were measured by quantitative real-time PCR (qPCR). Primers for all the genes examined in the study were either designed from available nucleotide sequences for each transcript from the NCBI database (http://www.ncbi.nlm.nih.gov/) using Oligo analyzer (http://eu.idtdna.com/analyzer/applications/oligoanalyzer/default.aspx) or previous literature (Table 3). A single peak from melting curve of each amplicon was examined to ensure specificity of the primers (Fig. S3).

**Table 3:** Primer pairs for quantitative real-time PCR (qPCR).

Gene	Source of sequence	Primer Sequence	Size (bp)	PCR Efficiencies
***Vitellogenin***	ABB89953	F: 5′-ATTCGGAACGTGCATTTGCTGCA-3′	188	96.2%
		R: 5′-GTTCTCAAGCATTGTGACAGGATT-3′		
***Dmc1***	Leelatanawit et al., 2008	F: 5′-ATGTGCGAGAAGCGAAGGC-3′	150	96.8%
		R: 5′-GCAGAGAGTGTGGGAGATTTGTG-3′		
***EF-1α***	Leelatanawit et al., 2008	F: 5′-TTCCGACTCCAAGAACGACC-3′	122	96.5%
		R: 5′-GAGCAGTGTGGCAATCAAGC-3′		
***GAPDH***	AI770197	F: 5′-ACATCGTTGAGTCCACTGGTGTGTT-3′	103	98.7%
		R: 5′-GCATCGGCAGAAGGAGCGG-3′		
***β-actin***	Qiu *et al*., 2008	F: 5′- GCCCTTGCTCCTTCCACTATC-3′	143	99.0%
		R: 5′- CCGGACTCTTCGTACTCATCCT-3′		
***18S rRNA***	Jarasrassamee *et al*., 2005	F: 5′-GAGACGGCTACCACATCTAAG -3′	182	97.6%
		R: 5′- ATACGCTAGTGGAGCTGGA-3′		

For construction of the standard curve for each transcript, a plasmid containing the transcript was constructed by cloning the PCR product of the transcript into a pGEM-T easy vector (Promega). The resulting vector was transformed into *E. coli* JM109. The plasmid was extracted and used as the template for construction of the standard curve by 10-fold serial dilutions (10^3^–10^8^ copy numbers).

Each qPCR reaction was performed in a 20 µl total reaction volume containing 2X iQ™ SYBR® Green Supermix (Bio-Rad), 200 ng of first strand cDNA template, and 0.2 µM of a primer pair. Cycling parameters were 95°C for 2.5 min; followed by 40 cycles of 95°C for 30 sec, 58°C for 20 sec, and 72°C for 30 sec. The specificity of PCR products was confirmed by melting curve analysis performed from 55°C–95°C with a continuous fluorescent reading with a 0.5°C increment. Expression levels of different sample groups were statistically tested by ANOVA followed by Tukey test (*P*<0.05).

### Data Analysis

The stability of expression levels of reference genes was evaluated by NormFinder [52] and geNorm [51]. The stability value of each candidate reference gene from each sample group was assessed separately by NormFinder and geNorm methods. The copy numbers of the four candidate housekeeping genes were calculated from the threshold cycle (Ct) obtained from qPCR experiment. These values were used as input to determine expression stability using the two software-based approaches. Moreover, relative expression levels of known ovary-relevant gene *Vitellogenin* (*Vg*) and known testis-relevant gene *Dmc1* were examined using each of the four housekeeping genes as a reference gene to compare with previously reported expression patterns to see the robustness of each candidate as a reference gene.

## Supporting Information

Figure S1 **Relative expression levels in term of copy numbers of a known testis-relevant marker,** ***Dmc1*** **, to the expression levels of the housekeeping genes in wild broodstock from Andaman sea black), wild broodstock from Gulf of Thailand (diagonal lines), 18-month-old domesticated shrimp (DS) (gray), 14-month-old DS (horizontal lines), 10-month-old DS (diamond), and 4-month-old DS (gray spots).** Different letters above the bars of each graph signify statistical differences in gene expression levels within the sample group.
(TIF)

Figure S2 **Relative expression levels in term of copy numbers of a known ovary-relevant marker,** ***Vitellogenin*** **(** ***Vg*** **), to the expression levels of the housekeeping genes in three ovary sample groups:** **(A) Wild broodstock (WB) from four different ovarian maturation stages, (B) Domesticated shrimp (DS) at 18-, 14-, 10-, and 4-month-old (C) Domesticated broodstock before the ablation (D0), and after the ablation for 1 (D1), 4 (D4), and 7 (D7) days.** Different letters above the bars of each graph signify statistical differences in gene expression levels within the sample group.
(TIF)

Figure S3 **Melting curves of qPCR amplicons in (A) testis samples and (B) ovary samples.**
(TIF)
