# Supplementary figures and images for: Validation of Reference Genes for Real-Time PCR of Reproductive System in the Black Tiger Shrimp
Source: PLoS One. 2012 Dec 28;7(12):e52677. doi: 10.1371/journal.pone.0052677 (PMC3532477; doi:10.1371/journal.pone.0052677)

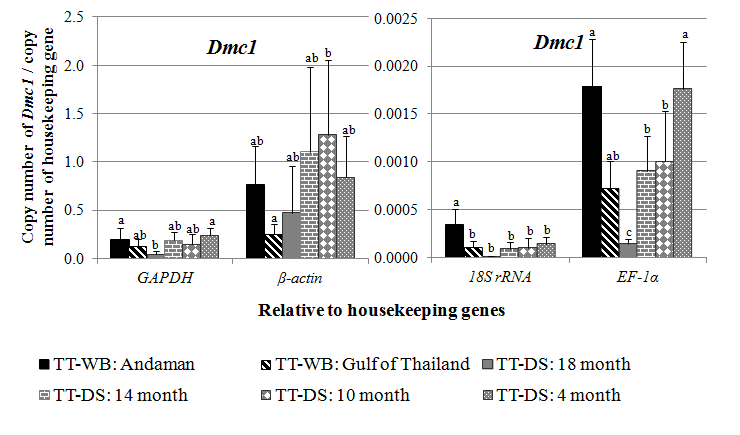

Supplement: Figure S1 — Relative expression levels in term of copy numbers of a known testis-relevant marker, Dmc1 , to the expression levels of the housekeeping genes in wild broodstock from Andaman sea black), wild broodstock from Gulf of Thailand (diagonal lines), 18-month-old domesticated shrimp (DS) (gray), 14-month-old DS (horizontal lines), 10-month-old DS (diamond), and 4-month-old DS (gray spots). Different letters above the bars of each graph signify statistical differences in gene expression levels within the sample group. (TIF) [file pone.0052677.s001.tif]

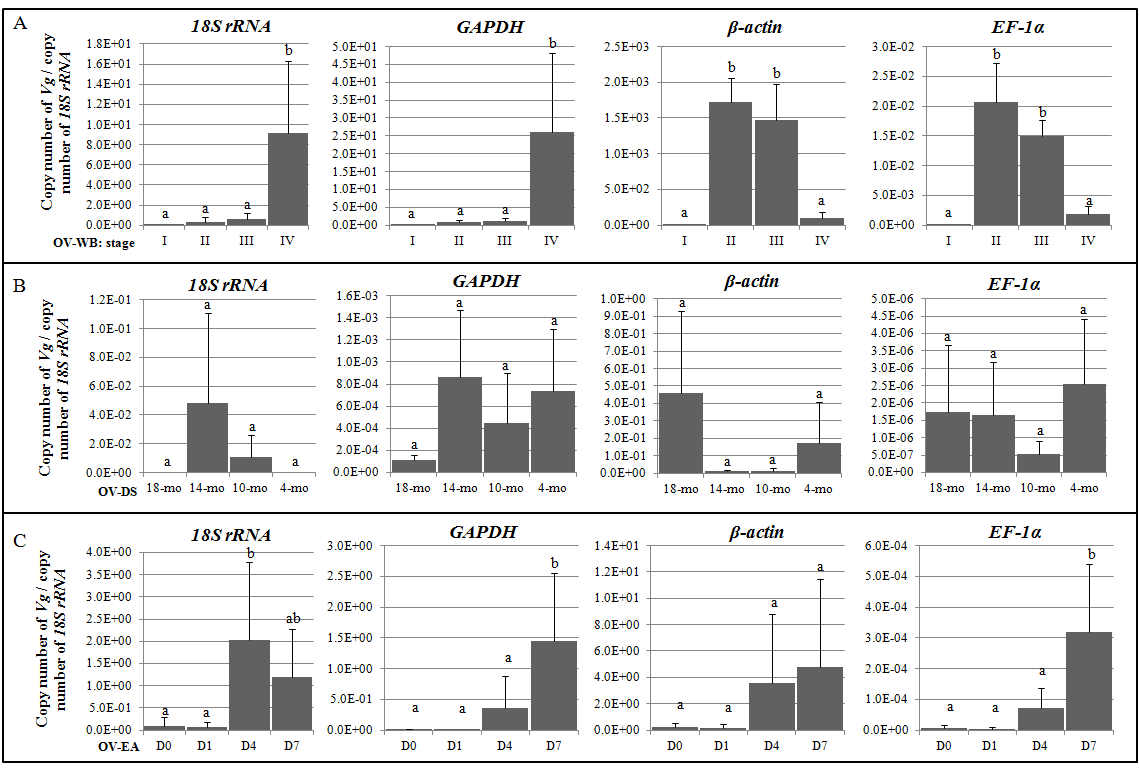

Supplement: Figure S2 — Relative expression levels in term of copy numbers of a known ovary-relevant marker, Vitellogenin ( Vg ), to the expression levels of the housekeeping genes in three ovary sample groups: (A) Wild broodstock (WB) from four different ovarian maturation stages, (B) Domesticated shrimp (DS) at 18-, 14-, 10-, and 4-month-old (C) Domesticated broodstock before the ablation (D0), and after the ablation for 1 (D1), 4 (D4), and 7 (D7) days. Different letters above the bars of each graph signify statistical differences in gene expression levels within the sample group. (TIF) [file pone.0052677.s002.tif]

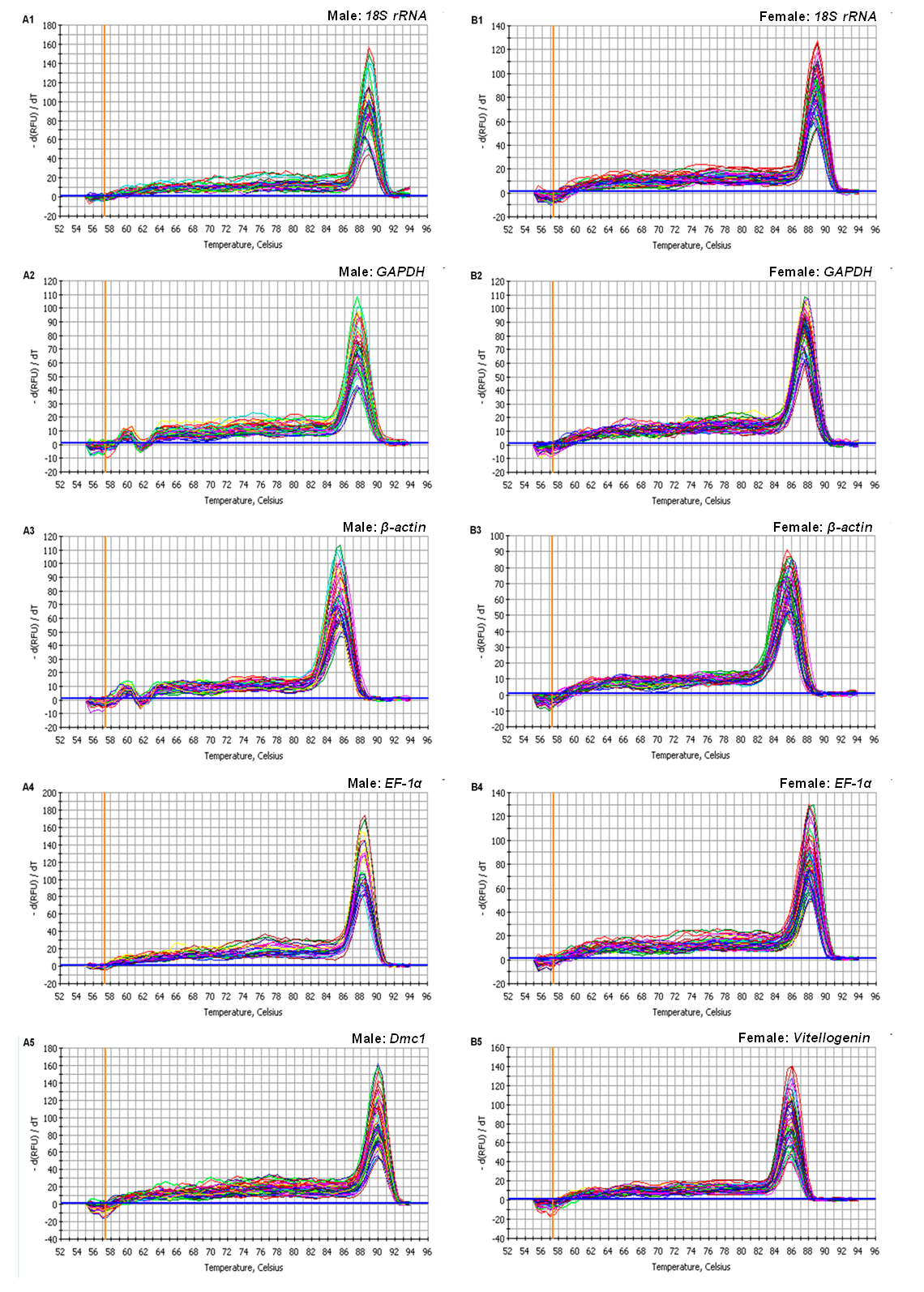

Supplement: Figure S3 — Melting curves of qPCR amplicons in (A) testis samples and (B) ovary samples. (TIF) [file pone.0052677.s003.tif]
